# Deep Learning-Driven Inversion Framework for Shear Modulus Estimation in Magnetic Resonance Elastography (DIME)

**Published:** 2025-12-15

**Authors:** Hassan Iftikhar, Rizwan Ahmad, Arunark Kolipaka

**Affiliations:** 1 Biomedical Engineering, The Ohio State University, Columbus, Ohio, USA; 2 Department of Radiology, The Ohio State University, Columbus, Ohio, USA; 3 Davis Heart & Lung Research Institute, The Ohio State University, Columbus, Ohio, USA

## Abstract

The Multimodal Direct Inversion (MMDI) algorithm is widely used in Magnetic Resonance Elastography (MRE) to estimate tissue shear stiffness. However, MMDI relies on the Helmholtz equation, which assumes wave propagation in a uniform, homogeneous, and infinite medium. Furthermore, the use of the Laplacian operator makes MMDI highly sensitive to noise, which compromises the accuracy and reliability of stiffness estimates. In this study, we propose the Deep-Learning driven Inversion Framework for Shear Modulus Estimation in MRE (DIME), aimed at enhancing the robustness of inversion. DIME is trained on the displacement fields-stiffness maps pair generated through Finite Element Modelling (FEM) simulations. To capture local wave behavior and improve robustness to global image variations, DIME is trained on small image patches. We first validated DIME using homogeneous and heterogeneous datasets simulated with FEM, where DIME produced stiffness maps with low inter-pixel variability, accurate boundary delineation, and higher correlation with ground truth (GT) compared to MMDI. Next, DIME was evaluated in a realistic anatomy-informed simulated liver dataset with known GT and compared directly to MMDI. DIME reproduced ground-truth stiffness patterns with high fidelity (r = 0.99, R^2^ = 0.98), while MMDI showed greater underestimation. After validating DIME on synthetic data, we tested the model in *in vivo* liver MRE data from eight healthy and seven fibrotic subjects. DIME preserved physiologically consistent stiffness patterns and closely matched MMDI, which showed directional bias. Overall, DIME showed higher correlation with ground truth and visually similar stiffness patterns, whereas MMDI displayed a larger bias that can potentially be attributed to directional filtering. These preliminary results highlight the feasibility of DIME for clinical applications in MRE.

## 1. INTRODUCTION

Biomechanical tissue properties are closely linked to physiological and pathological processes.^1^ Among these, stiffness has been recognized as a valuable biomarker for detecting various pathological states.^2^ Changes in tissue stiffness can indicate the presence of diseases such as fibrosis,^3,4,5^ tumors,^6,7^ or inflammatory conditions.^8^ Historically, physicians and clinicians have relied on palpation as a fundamental procedure to detect the stiffness changes.^9^ However, palpation applies to organs close to the body’s surface and cannot be applied to the deeper tissues, making it challenging to detect abnormalities non-invasively. Additionally, the diagnostic reliability of palpation is limited due to its subjective nature.

Magnetic Resonance Elastography (MRE)^10^ has emerged as a promising technique to estimate the stiffness of tissues non-invasively. MRE is a three-step process in which (1) mechanical waves are induced in the tissue of interest via an acoustic vibrator; (2) the propagation of wave in the tissue of interest is captured using phase-contrast magnetic resonance imaging^11,12^ with motion-encoding gradients synchronized with the external vibration; and (3) the wave images are processed to estimate the stiffness of the tissue by solving an inverse problem.^13^

Various inversion algorithms have been developed over time, including, but not limited to, variations of Direct Inversion (DI),^14,15^ Local Frequency Estimation (LFE),^16,17^ Multimodal Direct Inversion (MMDI),^18^ and Non-Linear Inversions (NLI).^19^ These methods assume that the tissue of interest is infinite, uniform, homogeneous, and isotropic. In practice, however, these assumptions do not hold in most tissues, leading to potentially inaccurate stiffness measurements and limiting the robustness of stiffness estimation. Accurate estimation of tissue stiffness is essential, as stiffness is not only a key biomarker for staging liver fibrosis, but also a confounded marker influenced by other histopathological conditions such as inflammation, portal hypertension, and hepatic venous congestion.^20^ Precise stiffness estimation, therefore, not only improves the reliability of fibrosis staging but also enables disease-specific interpretation, allowing physicians to better distinguish among multiple pathological processes using stiffness as a single surrogate biomarker.

Recently, deep learning (DL)-based methods have gained prominence for a variety of imaging tasks, including classification, segmentation, registration, and reconstruction.^21,22,23,24^ To address the above-discussed limitations in MRE inversion, Murphy et al.^25^ proposed a DL-based framework to estimate tissue stiffness from displacement wavefields; however, this work relied on simulated wavefields that were created by assuming homogeneous material properties throughout the geometry of the tissue, neglecting the local gradients in mechanical properties. Subsequently, Solamen et al.,^26^ demonstrated MRE displacement-to-elastogram mapping using Convolutional Neural Networks (CNNs), but the performance from their approach was limited by insufficient training diversity and lack of noise considerations. Scott et al.,^27^ attempted to address the homogeneity assumptions by introducing inhomogeneous material distributions using a coupled harmonic oscillator model. However, this dataset did not fully capture the elastic waves physics or boundary conditions essential for accurate stiffness estimation. Also, the use of curl as a preprocessing step is known to amplify the noise. Ma et al.^28^ introduced a dual-consistency framework and utilized Travelling Wave Expansion (TWE) model for dataset generation, but this required multifrequency and multidirectional MRE acquisitions, hence increasing the scan time while still relying on simplifying assumptions of tissue uniformity, linearity and homogeneity. Ragoza et al.^29^ proposed the utility of physics-informed neural networks to solve this inverse problem; however, the inversion still relied on Helmholtz inversion equation, which assumes tissue linearity and homogeneity. Zhang et al.^30^ utilized finite difference time domain method for synthetic data generation; however, their framework did not explicitly account for realistic boundary conditions. Recently, Bustin et al.^31^ proposed ElastoNet, a CNN–based inversion framework trained on synthetically generated 5×5 wave patches derived from point-source excitations, achieving frequency and resolution independent stiffness mapping under locally homogeneous, isotropic, and viscoelastic material assumptions. To overcome the inherent limitations of analytical MRE inversion, most notably their reliance on assumptions of tissue uniformity, homogeneity, isotropy, and an infinite medium, there remains a need for an inversion framework that can more accurately model realistic tissue behavior. DL offers a promising data-driven alternative. However, DL-based methods often require a large corpus of labeled training data. In many applications, including MRE, such ground-truth data is not available. Moreover, the performance of DL-based models is fundamentally constrained by the realism and quality of the training data, making accurately modeled datasets essential for reliable stiffness estimation. Although self-supervised and unsupervised learning strategies have been proposed to mitigate this dependency, they typically require de novo training for each dataset, which limits their clinical utility.^32^ In this work, we propose a **D**eep Learning-based **I**nversion framework for shear **M**odulus Estimation in MR**E (DIME)** that is designed to overcome these challenges while preserving clinical practicality. DIME introduces a pipeline for generating realistic paired displacement–stiffness data through FEM, capable of capturing spatially varying tissue mechanics that conventional simplified models fail to represent. The dataset is constructed in a patch-based format, motivated by the fact that stiffness is a local mechanical property, and the corresponding shear wavelength varies spatially with local stiffness. A CNN is then used to learn the mapping from complex-valued displacement field patches to their corresponding shear modulus. By training on patches rather than full images, DIME effectively learns localized wavelength–stiffness relationships, enabling accurate stiffness estimation at fine spatial scales, which is a key advantage of the framework. To evaluate the performance of DIME, we developed a highly realistic and innovative dataset, generated through advanced simulation techniques.^33^ This synthetic dataset not only captures the complexities of tissue behavior but also provides a robust foundation for assessing the performance of DIME against existing methods like MMDI. By using this carefully designed dataset, DIME is evaluated under conditions that closely approximate real-world tissue mechanics, where ground-truth stiffness is typically unknown, allowing direct performance assessment of both DIME and MMDI against known reference values. Once trained, DIME performs patch-wise stiffness estimation within milliseconds, making it computationally efficient and practically deployable.

## 2. THEORY

DIME formulates the problem of stiffness estimation as a supervised learning task, offering a data-driven alternative to analytical inversion approaches. Rather than relying on derivative-based formulations such as the Helmholtz equation, DIME directly learns the mapping between complex displacement wavefields and spatially varying stiffness distributions using a CNN $f_{\theta }$, parameterized by weights θ:

(1) $$f_{\theta }:\text{u}\to \mu$$

**Figure 1:**
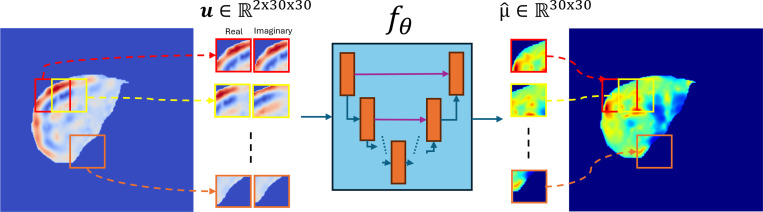
Overview of DIME Framework: Complex displacement fields are divided into 2×30×30 patches (real and imaginary channels) and processed by a neural network $f_{\theta }$ to predict corresponding stiffness patches $\mu \in {\mathbb{R}}^{30x30}$, which are then aggregated downstream in the pipeline into a full stiffness map.

Where $\text{u}\in {\mathbb{R}}^{2xHxW}$ contains two real-valued channels corresponding to the real and imaginary components of the complex displacement field, and $\mu \in {\mathbb{R}}^{HxW}$ is the shear modulus map. The high-level description of DIME is provided in Fig 1.

### 2.1. Neural Network Architecture

**Figure 2:**
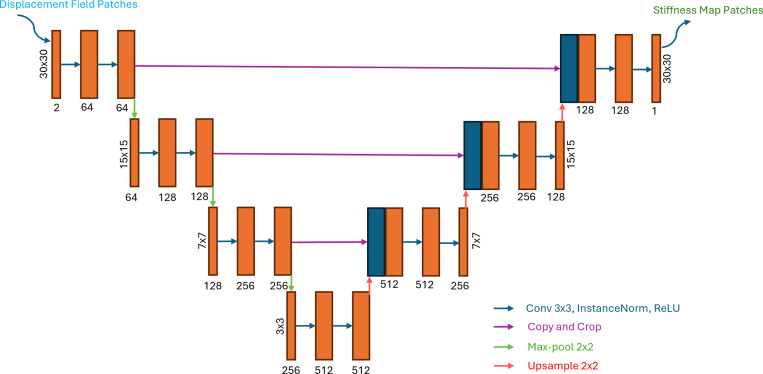
Model architecture

The proposed model $\left(f_{\theta }\right)$ is based on a U-Net, designed to map complex-valued displacement fields to shear modulus distributions. Each input is a 2×30×30 patch containing the real and imaginary components of the first harmonic displacement field, and the network outputs a corresponding 30×30 stiffness map. The network consists of four encoding and four decoding blocks, with symmetric skip connections between corresponding levels. Each convolutional block uses 3×3 kernels and Leaky ReLU activations, followed by batch normalization. The number of channels starts at 128 in the first layer and doubles at each subsequent block (i.e., 64 → 128 → 256 → 512), before symmetrically decreasing back to 1 in the final output layer. The architecture of the model is described in Fig 2.

### 2.2. Dataset Generation

To train $f_{\theta }$, we constructed a dataset of 900 synthetic phantoms generated via Finite Element Modeling (FEM) using COMSOL Multiphysics (COMSOL Inc., Massachusetts, USA). These phantoms were designed to encompass a wide range of anatomically and mechanically realistic patterns through variations in geometry, material properties, inclusion configurations, and boundary conditions.

**Figure 3:**
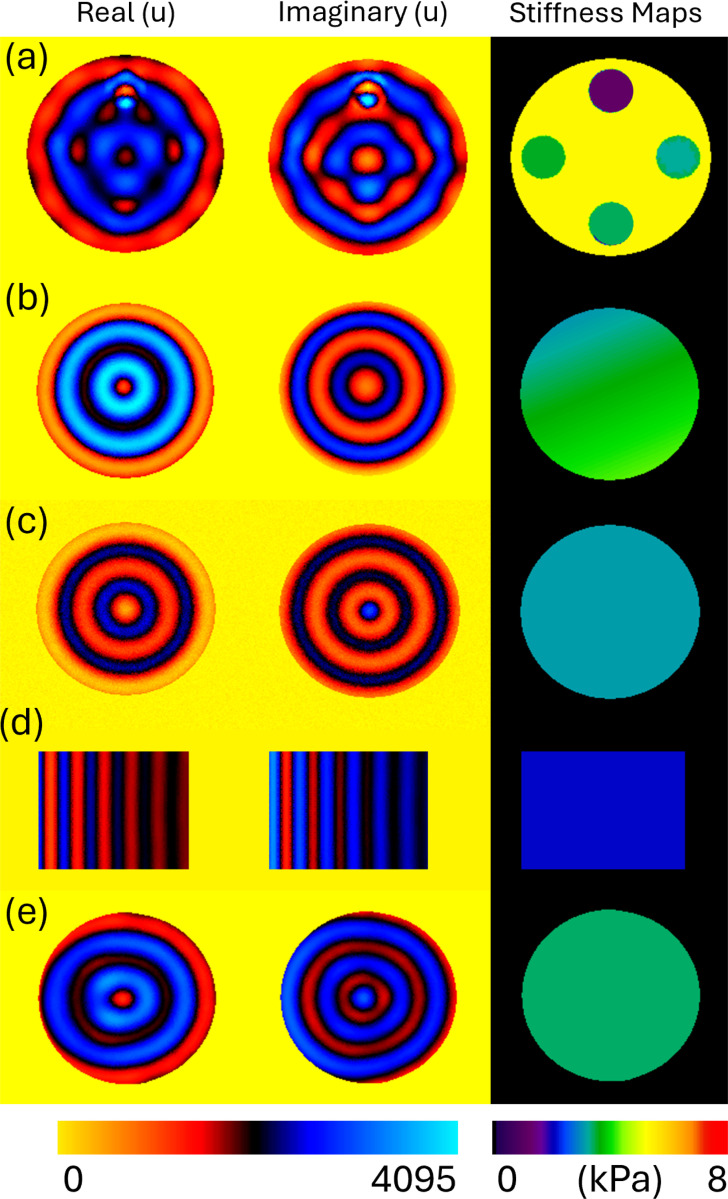
Simulated phantoms used for stiffness reconstruction. (a) Phantom with multiple circular inclusions of varying stiffness. (b) Phantom with linear stiffness variations. (c) Homogeneous circular phantom with centered driver. (d) Homogeneous square phantom (e) Homogeneous circular phantom with off-centered driver. Each set shows the real and imaginary components of displacement fields along with the corresponding stiffness map.

All simulations were performed with a Poisson’s ratio of 0.499 and a density of 1000 kg/m^3^. Planar wave propagation was induced by applying prescribed displacements ranging from 0.3 mm to 0.9 mm. Representative examples from key phantom classes are illustrated in Fig. 3. All material regions (including inclusions and background tissue) were assigned shear modulus values sampled from a uniform distribution of 1 to 8kPa and damping from 0.05 to 0.3. Circular phantoms were modeled in 3D and square phantoms in 2D, with mesh resolution adapted accordingly. The complete dataset composition is summarized in Table 1.

The complex-valued first harmonic displacement field was extracted as the first harmonic of the temporal Fourier transform of simulated phase images. Displacement and stiffness maps were then resampled to a uniform spatial resolution of 1mm and divided into 30×30 patches.

Each input patch contained two real-valued channels corresponding to the real and imaginary parts of the displacement field, while the output patch represented the shear modulus. Patches containing fewer than 50% non-zero pixels were excluded, resulting in 25,000 training, 5,000 validation, and 3,000 testing patches. Validation and test sets were drawn from the same distribution as the training data, without spatial overlapping. This patch-based approach improved sampling efficiency and enabled localized learning of wave-to-stiffness mappings across diverse anatomical and mechanical configurations. Fig 1 illustrates the patch-based approach.

### 2.3. Model Training

The model was trained using the Adam optimizer (learning rate 3×10^−4^, batch size 128) for 150 epochs on an NVIDIA RTX 3090 GPU. A step-based learning rate scheduler was used, with a decay factor (λ=0.8) applied every 20 epochs. The total training time was approximately 10 hours. Early stopping based on validation loss was used to prevent overfitting. The learning objective is to minimize the discrepancy between predicted and ground-truth stiffness maps over a dataset of FEM-generated samples, formulated as the following optimization problem:

(2) $$\theta^{*}=\text{arg}\;\underset{\theta }{\text{min}}\sum_{i=1}^{N}𝓛\left(f_{\theta }\left(u_{i}\right),\mu_{i}\right)$$

The composite loss function 𝓛 includes two components:

$$𝓛={𝓛}_{MSE}+\lambda .{𝓛}_{Total\;Variation}$$

The term ${𝓛}_{MSE}$ computes pixel-wise reconstruction error, and is defined as

$$L_{\text{MSE}}=\frac{1}{NCHW}\sum_{i=1}^{N}\sum_{c=1}^{C}\sum_{p=1}^{H}\sum_{q=1}^{W}{\left({\hat{\mu }}_{i}^{\left(c\right)}(p,q)-\mu_{i}^{\left(c\right)}(p,q)\right)}^{2}$$

Where N, C, H, and W denote the batch size, number of channels, image height, and width respectively. ${\hat{\mu }}_{i}^{\left(c\right)}(p,q)$ and $\mu_{i}^{\left(c\right)}(p,q)$ represent the ground truth and predicted shear modulus at spatial location (p,q) for channel c of i^th^ sample. The isotropic total variation term given by ${𝓛}_{Total\;Variation}^{iso}$ promotes spatial smoothness within each patch, discouraging spurious noise and artifacts without over-smoothing anatomical features. It is defined as:

$${𝓛}_{Total\;Variation}^{iso}=\sum_{i=1}^{N}\sum_{c=1}^{C}\sum_{p=1}^{H}\sum_{q=1}^{W}\sqrt{{\left(\nabla_{x}{\hat{\mu }}_{i}^{\left(c\right)}(p,q)\right)}^{2}+{\left(\nabla_{y}{\hat{\mu }}_{i}^{\left(c\right)}(p,q)\right)}^{2}+\epsilon }$$

Where $\nabla_{x}$ and $\nabla_{y}$ denote the forward finite differences along the horizontal and vertical directions, respectively, and $\epsilon =10^{-8}$ is a small constant added for numerical stability. The hyperparameter λ controls the strength of this regularization. The regularization coefficient λ balances fidelity and smoothness, helping patch-wise smoothness while preserving structural features relevant to anatomical variations. The code of DIME will be publicly available with some evaluation datasets upon publication at https://github.com/HassanIftikhar013/DIME.

## 3. METHODS

To evaluate the generalization capability of DIME, we designed a series of experiments using datasets distinct from the training set and arranged them in the order of increasing complexity. The goal was to assess how well a model trained on small synthetic patches generalizes to larger, full-sized phantoms and in vivo images using a patch-by-patch inference approach (Fig. 1). We first tested the model in homogeneous and heterogeneous digital phantoms to evaluate its accuracy in detecting varying stiffness levels. Next, we used a hybrid dataset by embedding anatomical heterogeneity from in vivo MMDI scans into new FEM simulations. Finally, we applied the trained model to in vivo liver MRE data to assess real-world performance. In all evaluations, we benchmarked DIME’s performance against MMDI, the current gold standard in stiffness reconstruction, to assess how well our data-driven approach compares to traditional inversion techniques.

### 3.1. Evaluation of DIME on simulated phantoms

#### (a) Homogeneous Phantom Evaluation:

To evaluate DIME on previously unseen data, we generated a new set of 50 homogeneous cylindrical phantoms specifically for testing. This dataset was distinct from the training set and was designed to assess the model’s ability to recover uniform stiffness values from simple geometries. The phantoms were simulated using the same FEM framework described in Section 2.2, with constant shear moduli sampled from 1 to 8 kPa. All other simulation parameters (e.g., damping, density, vibration frequency) matched those used during training. First harmonic displacement fields were extracted and divided into overlapping 20×20 patches (stride = 3). Patch-wise predictions were aggregated by averaging overlaps to reconstruct full-field stiffness maps.

For comparison, MMDI maps were computed on the same data. Post-processing involved Butterworth bandpass filtering (2–128waves/FOV) to remove longitudinal waves and directionally filtered to remove reflected waves. This evaluation provided a controlled baseline to benchmark DIME’s accuracy against MMDI under minimal spatial variation. Evaluation metrics included mean stiffness, inter-pixel standard deviation, and R^2^ relative to the known ground truth.

#### (b) Heterogeneous Phantom Evaluation:

To test the sensitivity of DIME to various stiffness regions in a geometry, we evaluated DIME on cylindrical phantoms containing four randomly placed inclusions. These phantoms were constructed using the same simulation framework as before, but with spatially varying material properties sampled from 1 to 8 kPa for both background and inclusions. Inclusion shapes, locations, and stiffness values were randomized across the dataset. Notably, this specific pattern of heterogeneity was not present in the training set, making it a strong test of generalization. As before, testing was performed in a patch-wise manner, and full stiffness maps were reconstructed by aggregating patch predictions.

Predicted maps were evaluated for their ability to localize inclusions, preserve structural boundaries, and recover regional stiffness values, with results benchmarked against MMDI. MMDI-based stiffness maps were calculated by applying the same post-processing as described in Section 3.1 (a).

### 3.2. Evaluation of DIME on anatomy-informed phantoms

To assess the performance of DIME on realistic and spatially heterogeneous cases, we developed a novel hybrid dataset that mimics the complexity of in vivo liver tissue while retaining access to ground-truth stiffness. This was achieved by first acquiring liver MRE data from 15 human subjects using a 1.5T clinical scanner (Aera, Siemens Healthineers, Erlangen, Germany), after obtaining IRB approval and written informed consent. Each subject was positioned in a head-first supine position inside the scanner, and an acoustic driver (Resoundant Inc., Rochester, MN) was used to generate the 60 Hz mechanical waves in the liver. A gradient echo-based MRE sequence^34,35^ was used to obtain shear waves in the axial slices of the liver. Imaging parameters included TR/TE of 25/20.8ms, field of view of 36×36cm^2^, matrix size of 128×64 with GRAPPA acceleration of 2, and four contiguous 5mm axial slices with motion encoding applied in the slice direction (duration: 16.7ms). Four temporal phase offsets were acquired per slice to capture the propagating wavefield. Wave images were processed using the MMDI algorithm, applying the same post-processing pipeline described in Section 3.1, including Butterworth bandpass filtering (2–128waves/FOV) and directional filter in 4 directions. The resulting stiffness maps were then smoothened using the polynomial fitting method proposed by Iftikhar et al.^33^ to generate continuous, spatially heterogeneous distributions.

These smooth stiffness maps were used as inputs for FEM simulations, which generated corresponding displacement fields. The resulting hybrid dataset allowed for a realistic evaluation of DIME’s performance on wavefields closely resembling in vivo data, while maintaining a known ground-truth stiffness distribution for quantitative comparison.

The final dataset consisted of 50 slices extracted from 15 subjects, with the FEM-generated displacement fields serving as the model input, and the polynomial-fitted MMDI stiffness maps serving as the ground truth outputs.

### 3.3. Evaluation of DIME on in vivo settings

For in vivo evaluation, we applied the trained DIME model directly to the acquired wave images from the 15 subjects described in Section 3.2. Unlike previous evaluations that relied on FEM-generated displacement data, this experiment assessed the model’s performance on wavefields acquired in vivo subjects. The displacement data was processed patch-by-patch using DIME to generate full-field stiffness maps. These predictions were then compared to the corresponding MMDI-derived stiffness maps, which served as the reference standard. To ensure consistency, MMDI maps were processed with the same post-processing pipeline described earlier in Section 3.2.

## 4. RESULTS

### 4.1. Validation of DIME on simulated phantoms

#### (a) Validation on Homogeneous Phantoms:

**Figure 4:**
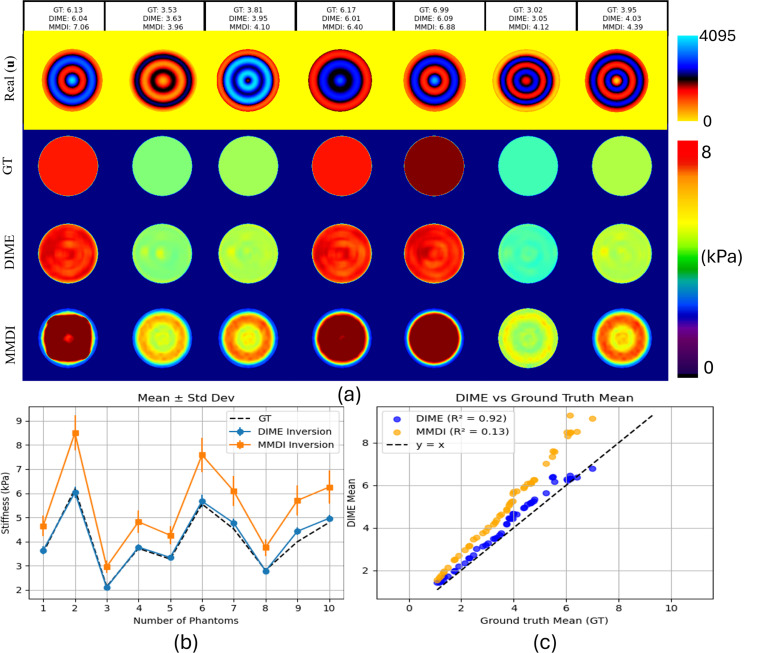
(a) Representative examples for Real wavefield components and corresponding stiffness maps reconstructed using DIME and MMDI. Mean stiffness values (in kPa) are shown above each column. **(b)** Mean ± standard deviation of predicted stiffness for DIME and MMDI compared to ground truth (GT) for 10 representative phantoms **(c)** Predicted vs. ground-truth mean stiffness for entire dataset; MMDI shows high bias, whereas DIME demonstrates reasonable alignment with ground truth.

Fig. 4 summarizes the reconstruction performance of DIME and MMDI across 50 simulated homogeneous phantoms. The real and imaginary components of the input wavefield are shown along with the predicted stiffness maps from both methods. Ground-truth (GT), DIME-derived, and MMDI-derived mean stiffness values (in kPa) are indicated. Quantitatively, DIME consistently produced stiffness maps with low inter-pixel variability and mean values closely aligned with ground truth. In contrast, MMDI reconstructions exhibited higher spatial variation and overestimated stiffness in most cases. The mean ± standard deviation across phantoms (Fig. 4b) shows that DIME maintained tight agreement with the true values, while MMDI showed larger deviations. Fig. 4c shows the correlation plot between DIME VS GT and MMDI vs GT. DIME achieved a high correlation with ground truth, while MMDI displayed a lower correlation and significant bias across the range of stiffness values.

#### (b) Validation on Heterogeneous Phantoms:

**Figure 5:**
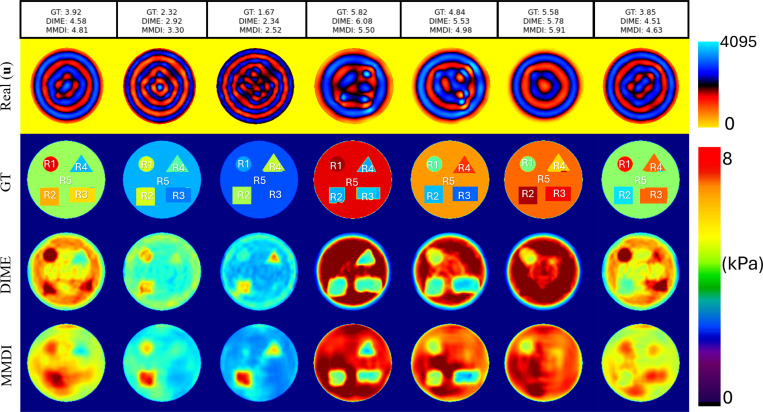
Evaluation of DIME on simulated heterogeneous phantoms. Representative examples of real wavefield components and the corresponding stiffness maps reconstructed using DIME and MMDI. Mean stiffness values (in kPa) are shown above each column.

Fig. 5 demonstrates a comparison of DIME and MMDI reconstructions on representative heterogeneous phantoms containing multiple inclusions of varying stiffness. Visual inspection indicated that DIME more accurately delineates inclusion boundaries and preserves structural contrast compared to MMDI, which often smooths transitions and introduces shape distortion. Across examples, DIME consistently localized inclusions and maintained sharper transitions between regions of varying stiffness.

**Figure 6:**
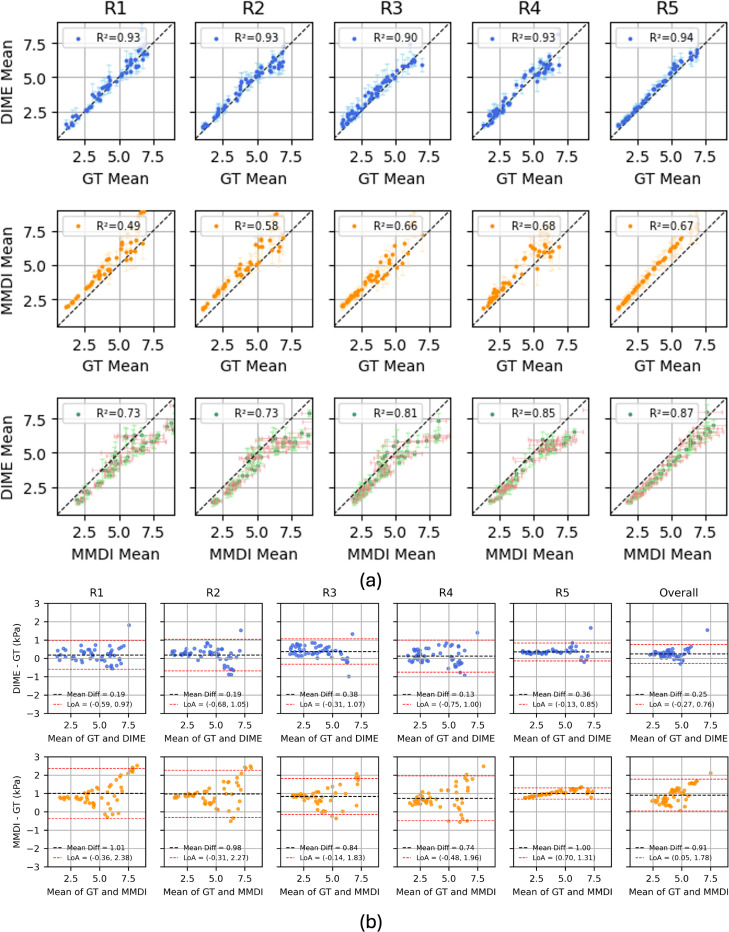
Region-wise correlation analysis across five test phantoms (R1–R5). Top row: DIME vs. ground truth (GT); Middle row: MMDI vs. GT; Bottom row: DIME vs. MMDI. DIME consistently shows stronger agreement with GT across all regions (R^2^ > 0.94), while MMDI exhibits lower accuracy and higher variability. **(b)** Bland–Altman plots comparing DIME (top) and MMDI (bottom) against ground truth across five regions and overall. DIME shows lower bias and tighter agreement limits than MMDI.

Quantitative evaluation is summarized in Fig. 6a, which reports region-wise mean and standard deviation comparisons across methods. DIME showed strong agreement with ground-truth. In contrast, MMDI exhibited systematic overestimation, especially in high-stiffness inclusions, with greater intra-region variance. DIME showed more stable predictions across background and inclusion regions, indicating better generalization to complex spatial distributions. Bland–Altman analysis (Fig. 6b) further supports the comparative evaluation of DIME and MMDI. When compared against ground truth, DIME exhibited narrower limits of agreement (−0.27 to 0.76kPa) and a smaller bias of 0.25kPa. In contrast, MMDI showed wider limits of agreement (0.05 to 1.78kPa) and a larger bias of 0.91kPa when estimating the overall mean stiffness of the phantoms. Similar trends were observed in region-wise analysis, where individual ROIs reinforced that MMDI consistently overestimated stiffness, particularly in regions with higher ground-truth stiffness values, indicating a systematic bias that was not seen in DIME.

### 4.2. Validation of DIME Anatomy Informed Phantoms

**Figure 7:**
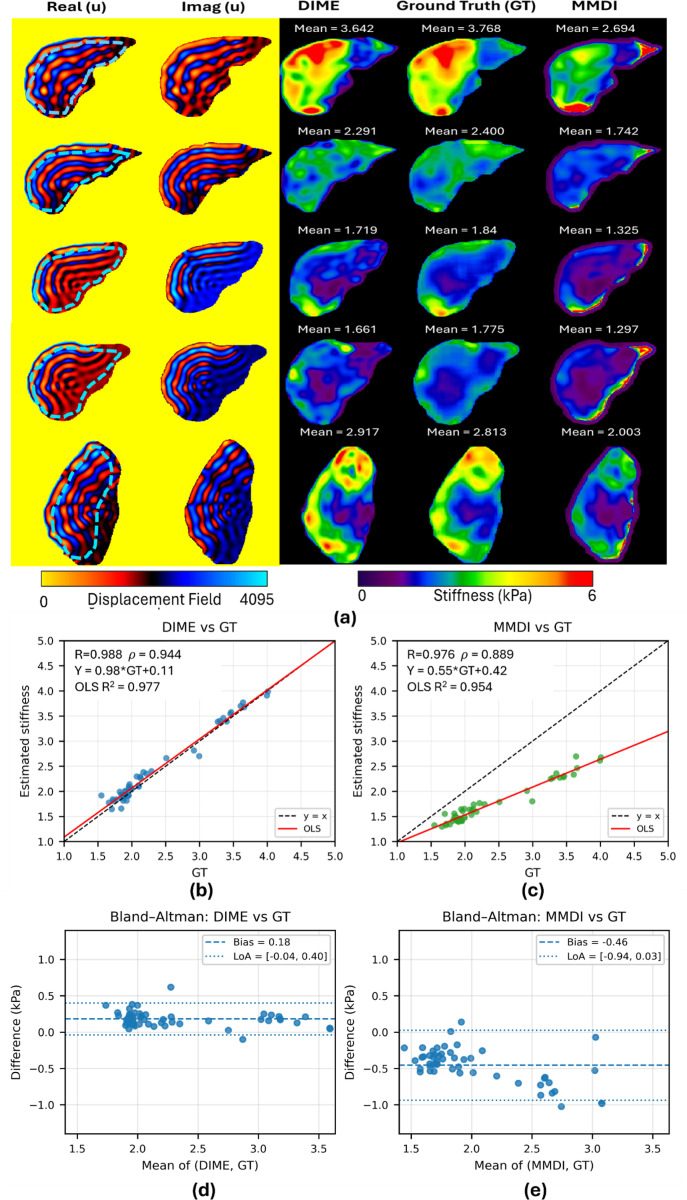
Evaluation of DIME on anatomy informed simulated phantoms. **(a)** Representative examples showing the real and imaginary components of the displacement field, together with stiffness maps reconstructed using DIME, MMDI, and Ground Truth (GT). Mean stiffness values are calculated within a region of interest (ROI) defined by the 95% confidence interval, with the ROI boundary indicated on the real displacement field. **(b–c)** Statistical comparisons of DIME and MMDI against GT, including multiple correlation coefficients. **(d–e)** Bland–Altman analysis illustrating the bias and limits of agreement for MMDI vs GT and DIME vs GT, respectively.

To assess reconstruction accuracy, representative slices from the simulated liver dataset are shown in Fig. 7(a), including the real and imaginary displacement fields and the corresponding stiffness maps from DIME, MMDI, and GT. Visual inspection shows that DIME closely replicates the spatial stiffness patterns observed in GT, whereas MMDI tends to underestimate stiffness under the same reconstruction conditions. Quantitative analysis supports these findings. As shown in Fig. 7(b), DIME demonstrates a strong linear correlation with GT, with a Pearson’s correlation of r=0.988, Spearman’s correlation of ρ=0.944, and an ordinary least squares (OLS) coefficient of determination $\left(R^{2}=0.977\right)$, with data points closely aligned along the identity line (y=x). In comparison, Fig. 7(c) shows that MMDI also correlates with GT (r=0.976,ρ=0.889), but with a lower regression slope (0.55) and a smaller OLS $R^{2}=0.954$, indicating systematic underestimation. Bland–Altman analysis (Figs. 7(d–e)) further highlights these differences. DIME exhibits a small bias of 0.18kPa and narrow limits of agreement (−0.04kPa, 0.4kPa), indicating close agreement with GT. Conversely, MMDI shows a larger negative bias (−0.46kPa) and wider limits of agreement (−0.94kPa, 0.03kPa), consistent with the observed underestimation.

### 4.3. Validation of DIME *in vivo*

**Figure 8:**
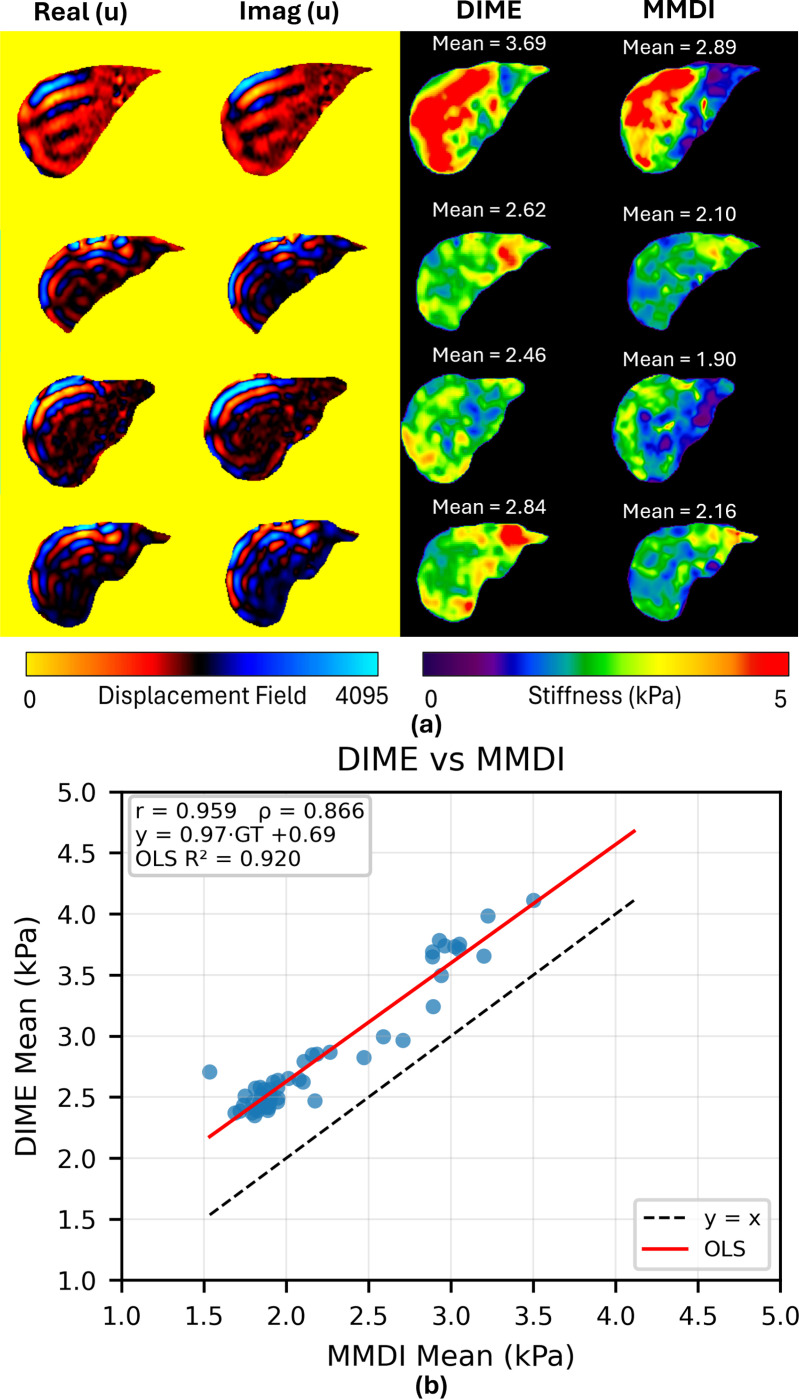
Reconstruction results of DIME and MMDI for in vivo liver data. **(a)** Input in vivo wave images (left) with corresponding stiffness maps for DIME and MMDI (right), annotated with mean ROI stiffness values. **(b)** Scatter plot comparing DIME and MMDI estimates, showing strong agreement with Pearson’s correlation coefficient (r=0.959) and Spearman’s correlation coefficient (ρ=0.866). The regression line is given by y=0.97x+0.69.

Representative stiffness maps reconstructed from in vivo liver MRE data of five volunteers are shown in Fig. 8a. Visual inspection indicates that both DIME and MMDI capture comparable spatial patterns of stiffness within the liver. Quantitative comparison across 50 liver slices further supports this observation. As shown in Fig. 8b, DIME and MMDI demonstrated strong agreement, with Pearson’s correlation coefficient of r=0.959 and Spearman’s correlation coefficient of ρ=0.866. Linear regression analysis yielded a fit of y=0.97x+0.69, with a slope close to unity, indicating strong linear agreement between the two methods. However, the positive intercept suggests the presence of a bias, consistent with a tendency of MMDI to slightly underestimate stiffness relative to DIME.

## 5. DISCUSSIONS

In this work, we introduced DIME, a DL–based inversion framework that leverages FEM-generated wavefields for stiffness reconstruction. Our results across multiple validation studies demonstrated that DIME recovers stiffness maps with higher fidelity to ground truth compared to the commonly used MMDI. Importantly, the evaluation encompassed both simple phantoms and anatomy informed FEM generated liver phantoms, as well as in vivo liver MRE, allowing for a broad assessment of performance.

### 5.1. Comparison of DIME and MMDI on simple simulated phantoms

In homogeneous phantom experiments, DIME maintained close agreement with ground truth, while MMDI consistently overestimated stiffness. Although both methods showed correlation with true values, MMDI displayed systematic bias. In heterogeneous phantoms, DIME more accurately delineated sharp stiffness boundaries and produced smoother reconstructions, consistent with the loss function’s penalization of inter-pixel variability. Region-based analysis revealed that DIME achieved higher $R^{2}$ values relative to ground truth across all ROIs, supported by Bland–Altman analysis showing narrower limits of agreement. By contrast, MMDI exhibited greater variability and a consistent bias toward overestimation. These findings suggest that DIME is particularly well-suited for detecting localized regions of elevated stiffness, such as those associated with tumors, where precise boundary recovery is clinically important.

### 5.2. Comparison of DIME and MMDI on anatomy informed phantoms

The anatomy-informed phantom study provided an intermediate step between simple phantoms and in vivo data, preserving ground-truth accessibility while introducing spatial heterogeneity. In this setting, DIME again demonstrated higher agreement with the ground truth, reflecting higher R^2^ values and reduced bias compared to MMDI. While MMDI achieved reasonable Pearson and Spearman correlations, the presence of bias lowered its overall accuracy. An interesting trend emerged: while MMDI overestimated stiffness in simple phantom experiments, it underestimated stiffness in anatomy informed cases. This discrepancy likely arose from the increased structural complexity of the waves in the anatomically realistic stiffness distributions. These results demonstrate DIME’s ability to accurately recover global stiffness under idealized, spatially heterogeneous conditions, outperforming the conventional MMDI. To the best of our knowledge, this study represents one of the first efforts where anatomically realistic heterogeneous stiffness maps were used to benchmark DL–based inversion against conventional MMDI. This setting provides a rare opportunity to assess reconstruction performance under realistic wave propagation conditions while retaining access to ground truth stiffness. Such conditions are seldom available in existing datasets, making this comparison particularly valuable.

### 5.3. Comparison of DIME and MMDI on *in vivo* liver MRE

For *in vivo* liver MRE data, the overall trend was consistent with the phantom studies. Both DIME and MMDI produced stiffness maps that captured the main spatial patterns and showed strong correlation. However, MMDI tends to underestimate the stiffness values compared to DIME, like the trend observed in FEM-generated anatomy informed phantom.

A similar bias pattern was also reported previous AI-based inversion studies,^27^ where AI-based reconstructed stiffness maps exhibited systematic bias relative to direct inversion results. However, this bias was not analyzed in depth, likely due to the absence of quantitative ground-truth stiffness values. This underscores the importance of having access to realistic labeled datasets, as demonstrated in our Study 3.2, which enabled a controlled evaluation and direct comparison between the conventional MMDI and the proposed DIME framework.

In our observation, this bias in MMDI inversion is likely linked to the use of directional band-pass filtering as a preprocessing step. The choice of cutoff frequencies in this filtering step (usually set between 2 and 128 waves per field of view in clinical liver MRE) has a strong influence on the quantitative outcome. While these cutoff values are commonly used in clinical practice, they may not necessarily be optimal and can introduce bias by affecting the absolute stiffness values. Although changing these parameters does not affect the overall spatial appearance of the maps, it can shift the absolute stiffness values and introduce bias. In contrast, DIME does not rely on such parameter tuning, which makes its estimates more stable and less sensitive to filtering choices.

### 5.4. Limitations

#### 5.4.1. Noisy Measurements

In this study, in vivo liver MRE data exhibited relatively low noise levels. While noise was artificially introduced into the phantom training data to encourage generalization, a systematic evaluation of DIME and MMDI performance across varying noise levels was not conducted. Future work will include controlled experiments at different noise intensities to better characterize robustness.

#### 5.4.2. Network Architecture

A U-Net–based architecture was adopted as a baseline. However, other network designs that have demonstrated success in medical imaging could be explored. Evaluating advanced architectures, such as attention-based networks or physics-driven loss functions,^26^ may further improve inversion performance and generalizability.

#### 5.4.3. Other directions of wave propagation

The current model was trained on slice-by-slice phantom data using only in-plane wave information. This restriction could lead to biased stiffness estimation in complex geometries. Extending the framework to fully 3D MRE acquisitions, where both in-plane and through-plane displacement fields are available, would allow for more robust stiffness reconstruction. Such an extension would also broaden the applicability of DIME to challenging domains such as cardiovascular and cardiopulmonary MRE.

#### 5.4.4. Applications

This work focused on liver MRE, an established clinical application for staging fibrosis. While this provided a clinically relevant test case, the DIME framework is not limited to hepatic applications. Future studies will adapt the pipeline to other organs in different disease states, incorporating 3D data, higher noise levels, and diverse wave propagation patterns.

#### 5.4.5. Study Population

Validation was performed on a limited cohort of patients with fibrotic liver conditions. Larger-scale clinical studies are needed to evaluate DIME’s reliability across different stages of fibrosis and to confirm its potential for integration into routine clinical practice.

## 6. CONCLUSIONS

In this study, we introduced DIME, a DL–based inversion framework trained on FEM-generated datasets to enable robust estimation of tissue stiffness. The proposed pipeline provided a systematic comparison against the conventional clinical MMDI approach across multiple evaluation settings. In both simple and heterogeneous digital phantoms, DIME demonstrated stronger correlations with ground truth. For anatomy informed phantoms, DIME preserved fine spatial detail and achieved higher correlations with ground truth relative to MMDI. Finally, preliminary in vivo liver MRE experiments indicated that DIME could generate reliable stiffness maps while mitigating some limitations inherent to MMDI. Collectively, these findings highlight the potential of DIME as a complementary inversion technique for clinical MRE, warranting further validation in larger and more diverse patient cohorts.

## Figures and Tables

**Table 1:** Specifications of FEM-based simulations

Phantom Type	Dim.	Count	Description	DOF

Homogeneous cylindrical phantoms (Fig. 3c)	3D	400	Uniform stiffness, centered planar wave excitation	248,205
Cylindrical phantom with linear stiffness variations (Fig. 3b)	3D	100	Linearly varying μ, with centered excitation	248,205
Cylindrical phantoms with off-centered excitation (Fig. 3e)	3D	100	Uniform μ, with asymmetric boundary displacement	457,269
Cylindrical phantoms with 4 fixed-position inclusions (Fig. 3a)	3D	50	Four inclusions with fixed locations; varying stiffness and damping	225,588
Cylindrical phantoms with 2 random inclusions placement	3D	50	Two inclusions placed randomly per phantom	252,801
Homogeneous square phantoms (Fig. 3c)	2D	100	Uniform stiffness, planar wave propagation	25,530
Square phantoms with 4 random inclusions	2D	100	Four inclusions per phantom, randomly positioned and parameterized	34,018
